# Overlooked Holmes’ clinical signs: reevaluation by recent physiological findings

**DOI:** 10.1186/s40673-015-0033-z

**Published:** 2015-11-06

**Authors:** Takahiro Ishikawa, Shinji Kakei, Hiroshi Mitoma

**Affiliations:** Tokyo Metropolitan Institute of Medical Science, Tokyo, Japan; Department of Medical Education, Tokyo Medical University, Tokyo, Japan

**Keywords:** Cerebellar symptoms, Ataxia, Asthenia, Adventitious movement, Cerebellar motor control

## Abstract

Holmes proposed not only the term ataxia, but also opposite clinical signs related to muscle recruitment, which have escaped clinical attention; (*1*) *asthenia*, representing delay in initiating muscle contraction and slowness in attaining exertion of full power, and (*2*) *adventitiousness*, representing adventitious movements. Recent physiological studies have shown that cerebellar outputs are modified by release or facilitation of Purkinje cell-mediated inhibition on dentate neurons. We believe that asthenia and adventitiousness, which correlate with deficits in the control of disinhibition and inhibition, respectively, deserve more attention in clinical examination.

*Sir,*

**Fig. 1:**
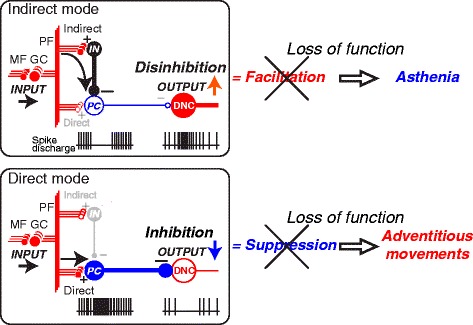
Relationship between breakdown of two modes of dentate nucleus cell (DNC) output and asthenia (top) and adventitious movements (bottom). *Asthenia* (*top*) is the consequence of breakdown of the *Indirect mode* while *Adventitious movements* (*bottom*) is the result of breakdown of the *Direct mode*. The diagram provides a summary of the functional organization of the cerebellum (Ishikawa et al. [5]). In the cerebellar cortex, mossy fiber (MF) inputs (INPUT) are relayed by granule cells (GCs) and processed through two parallel but different pathways, an indirect pathway (Indirect) and a direct pathway (Direct). In the indirect pathway (*top*), parallel fiber (PF) inputs activate interneurons (INs) that suppress PCs. Because PC activity provides tonic suppression of DNCs, suppression of PC activity facilitates DNCs through disinhibition (OUTPUT↑). Breakdown of this output mode leads to a decrease in facilitatory output, resulting in *Asthenia*. In the direct pathway (*bottom*), PF inputs excite PCs directly. Because PCs are inhibitory, their activation suppresses the DNCs (OUTPUT↓). Breakdown of this output mode leads to a decrease in suppression, resulting in *Adventitious movements*. CF: climbing fiber. (+): excitatory synapses, (−): inhibitory synapses

In 1917, Holmes proposed the term ataxia, which included decomposition of movement, asynergia, dysmetria, and tremor, as a clinical sign of cerebellar disorders [1]. However, *asthenia* and *adventitiousness*, both of which were also described in the original publication, have been clinically overlooked in spite of their clinical significance. Recently, these clinical signs were reevaluated in a consensus paper by Bodranghien et al. (chapter by Steiner and Timmann) [2]. The present letter proposes other underlying physiological mechanisms of these overlooked clinical signs.

Holmes also described other curious complaints by the patients “they have not nearly so much power in them (affected limbs)” (page 469). Then he described “a delay in initiating muscular contractions and a slowness in attaining the exertion of the full power” (page 474–5), which was defined clinically as *asthenia* [1]. The concept of *asthenia* was originally proposed by Luciani based on animal experiments [3, 4]. Luciani described a deficit in muscular force production during voluntary movements in hemicerebellectomized dogs and primates [3]. Thus, Holmes confirmed the occurrence of *asthenia* in patients and, furthermore, introduced a simple method for examination of *asthenia*; “when the observer’s hands are placed in the patient’s, and he is asked to grasp them firmly at a given signal; the slowness of the affected limb in starting the action and in developing full power is often unmistakable” (page 471) [1]. In addition, *adventitious movements* were described in a chapter on adiadochokinesis. Holmes did not only describe inability to execute alternate movements quickly and correctly, but also stressed *adventitious movements* at elbow and shoulder (page 486–7), which led to failure of fixing the proximal muscles to preserve correct posture [1]. Thus, *asthenia* and *adventitiousness* are opposite deficits of activities of a group of muscles. *Asthenia* represents failure of augmentation in muscles to be recruited, whereas *adventitious movement* is exaggerated activation of muscle to be paused. These clinical signs related to muscle recruitment could have been overlooked by clinicians, compared with ataxic symptoms; conspicuous deficits observed during multi-joint movements.

However, recent physiological findings explain well and systematically the two signs of *asthenia* and *adventitiousness*. We (TI and SK) found that during wrist movement of monkeys, a large proportion of Purkinje cells (PCs), with somatosensory receptive fields (RFs) in the distal arm, was strongly suppressed before movement onset, while the majority of dentate cells (DNs) with the same RFs showed concurrent burst of activity. In contrast, PCs with RFs in the proximal arm demonstrated marked and simultaneous increase in activity, while DNs with the same RFs were strongly suppressed [5]. Our observation suggests that activation of DNs generated by reduced inhibition from PCs, i.e., disinhibition, facilitates the execution of wrist movement, while suppression of the DNs by increased PC activity contributes to the stabilization of proximal muscles and improves task performance. Thus, deficits of disinhibition and inhibition of DNs could be the physiological counterparts of *asthenia* and *adventitiousness,* respectively (Fig. 1).

In conclusion, it seems that *asthenia* and *adventitiousness* reflect deficits in the control of disinhibition and inhibition that determines the strength of cerebellar outputs. Thus, these Holmes’ overlooked clinical signs could be clinically utilized as elements underlying ataxia.
